# Natural Disasters Intensity Analysis and Classification Based on Multispectral Images Using Multi-Layered Deep Convolutional Neural Network

**DOI:** 10.3390/s21082648

**Published:** 2021-04-09

**Authors:** Muhammad Aamir, Tariq Ali, Muhammad Irfan, Ahmad Shaf, Muhammad Zeeshan Azam, Adam Glowacz, Frantisek Brumercik, Witold Glowacz, Samar Alqhtani, Saifur Rahman

**Affiliations:** 1 Department of Computer Science, COMSATS University Islamabad, Sahiwal Campus, Sahiwal 57000, Pakistan; muhammadaamir@cuisahiwal.edu.pk (M.A.); ahmadshaf@cuisahiwal.edu.pk (A.S.); 2 Electrical Engineering Department, College of Engineering, Najran University Saudi Arabia, Najran 61441, Saudi Arabia; miditta@nu.edu.sa (M.I.); srrahman@nu.edu.sa (S.R.); 3 Department of Computer Science, Bahauddin Zakariya University, Multan 66000, Pakistan; emersonian194@gmail.com; 4 Department of Automatic Control and Robotics, Faculty of Electrical Engineering, Automatics, Computer Science and Biomedical Engineering, AGH University of Science and Technology, al. A. Mickiewicza 30, 30-059 Kraków, Poland; adglow@agh.edu.pl (A.G.); wglowacz@agh.edu.pl (W.G.); 5 Department of Design and Machine Elements, Faculty of Mechanical Engineering, University of Zilina, Univerzitna 1, 010 26 Zilina, Slovakia; frantisek.brumercik@fstroj.uniza.sk; 6 College of Computer Science and Information Systems, Najran University, Najran 61441, Saudi Arabia; smalqhtani@nu.edu.sa

**Keywords:** deep learning, natural disasters intensity and classification, convolutional neural network

## Abstract

Natural disasters not only disturb the human ecological system but also destroy the properties and critical infrastructures of human societies and even lead to permanent change in the ecosystem. Disaster can be caused by naturally occurring events such as earthquakes, cyclones, floods, and wildfires. Many deep learning techniques have been applied by various researchers to detect and classify natural disasters to overcome losses in ecosystems, but detection of natural disasters still faces issues due to the complex and imbalanced structures of images. To tackle this problem, we propose a multilayered deep convolutional neural network. The proposed model works in two blocks: Block-I convolutional neural network (B-I CNN), for detection and occurrence of disasters, and Block-II convolutional neural network (B-II CNN), for classification of natural disaster intensity types with different filters and parameters. The model is tested on 4428 natural images and performance is calculated and expressed as different statistical values: sensitivity (SE), 97.54%; specificity (SP), 98.22%; accuracy rate (AR), 99.92%; precision (PRE), 97.79%; and F1-score (F1), 97.97%. The overall accuracy for the whole model is 99.92%, which is competitive and comparable with state-of-the-art algorithms.

## 1. Introduction

Natural disasters are inevitable, and the occurrence of disasters drastically affects the economy, ecosystem and human life. Buildings collapse, ailments spread and sometimes natural disasters such as tsunamis, earthquakes, and forest fires can devastate nations. When earthquakes occur, millions of buildings collapse due to seismological effects [1]. Many machine learning approaches have been used for wildfire predictions since the 1990s. A recent study used a machine learning approach in Italy. This study used the random forest technique for susceptibility mapping of wildfire [2]. Floods are the most devastating natural disaster, damaging properties, human lives and infrastructures. To map flood susceptibility, an assembled machine learning technique based on random forest (RF), random subspace (RS) and support vector machine (SVM) was used [3]. As the population is growing rapidly, people need to acquire land to live on, and as a result the ecosystem is disturbed horrifically, which causes global warming and increases the number of natural disasters. Populations in underdeveloped countries cannot afford damages disasters cause to infrastructures. The aftermath of disasters leaves the humans in miserable situations, and sometimes the devastating effects cannot be detected; additionally, rescue operations cannot take place in most of the places and victims are unable to be identified due to geographical factors of the different areas. Disasters such as forest fires spread rapidly in dense areas, so firefighting is difficult to carry out; in this case, development of the strategy to predict such circumstances is crucial so that such disasters can be prevented beforehand. 

As the technologies are continuously improving, aviation systems have begun adopting smart technologies to develop unmanned aerial vehicles (UAVs) equipped with cameras, which can reach distant areas to identify aftereffects of natural disasters on human life, infrastructure, and transmission lines by capturing images and videos. Data acquired from these UAVs helps to identify the facial expressions of victims, the intensity of their situation and their needs in a post disaster scenario. It helps to take actions and carry out necessary operations to tackle devastating scenarios. Raw images obtained from camera-equipped UAVs are processed and neural network-based feature extraction techniques are applied to analyze the intensity.

A deep learning method for the reconstruction of two-dimensional cardiac magnetic resonance images was proposed to enhance the image data acquisition process. Cascade deep convolutional neural networks use a 10-fold method to reconstruct the feature map for the MR images. In this way, feature extraction sequence becomes very fast and it takes less than 5 to 10 s to extract the feature matrix [4].

Neural networks provide multilevel network architectures, where Convolutional Neural Networks (CNNs) are the most frequently implemented architecture as the direct input of multidimensional vector images, speech recognition, and image processing can be carried out with low complexity. CNNs efficiently perform feature extraction by denoising the images and removing interference and achieve highly accurate results [5].

The proposed multilayered deep convolutional neural network method works in two blocks of convolutional neural networks. The first block, known as Block-I Convolutional Neural Network (B-I CNN), detects the occurrence of a natural disaster and the second one, known as Block-II Convolutional Neural Network (B-II CNN), defines the intensity of the natural disaster. Additionally, the first block consists of three mini convolutional blocks with four layers each and includes an image input and fully connected layers. On the other hand, the second block also consists of three mini convolutional blocks with two layers each, including an image input layer and fully connected layer.

The remaining paper is divided into four sections: Section 2, describes the related work. Section 3 presents the methodology which elaborates on the proposed technique. The results and discussion are presented in Section 4 to explore the overall research outcomes and describe the used dataset. Finally, the proposed work is concluded in Section 5.

## 2. Related Work

Studies analyzing the intensity of natural disasters have gained significant attention in the current decade. A. Ashiquzzaman et al. [6] utilized a video source for fire detection; processing video sources is a feasible task due to convolutional neural networks (CNNs), which require high performance computational resources including graphics hardware, and thus a smart and cost-effective fire detection network is proposed based on architecture of convolutional neural networks. 

In convolutional neural networks, a model to detect wildfire smoke named wildfire smoke dilated dense net was proposed by Li et al. [7], consisting of a candidate smoke region segmentation strategy using an advanced network architecture. Mangalathu et al. [8] performed an evaluation of building clusters affected by earthquakes by exploring the deep learning method, which uses long short-term memory.

Natural disasters are unpredictable events, Hartawan et al. [9] enhanced multilayer perceptron algorithm by including convolutional neural network implemented on raspberry pi to find out the victims of natural disasters using streaming cameras and to aid the evacuation team to rescue the disaster victims. Amit et al. [10] proposed applying automatic natural disaster detection to a convolutional neural network using the features of disaster from resized satellite images of landslide and flood detections. Aerial images are able to show more specific and wider surface area of the ground, which helps acquire a vast amount of information about the occurrence of disaster. 

Social media networks such as Twitter where people share their views and information have been used as data sources to carry out disaster analysis. S. Yang et al. [11] used the information related to earthquake shared by users on Twitter as a dataset and input it to the real time event detection system based on convolutional neural networks. Implementation of a CNN module made it possible to successfully achieve the detection of an earthquake and its announcement by the government beforehand using information-based tweets. As the tweets provide a significant amount of information, Madichetty et al. [12] implemented a convolutional neural network to perform feature extraction on informative as well as noninformative tweets, categorizing dataset containing tweets by an artificial neural network. 

Social media is considered as a main source of big data, with data shared in the form of images, videos and text; after the occurrence of a disaster, social platforms are overflowed with different sorts of information which helps response teams to rescue the victims. The majority of the data contain ambiguous contents which makes it difficult for the rescue teams to make the right decisions. Nunavath et al. [13] reviewed previous research based on convolutional neural networks using social media as a dataset and efficiently analyzed the effectiveness of big data from social media during disaster management.

Using the two-layer architecture of a convolutional neural network (CNN), an efficient feature extraction method was applied to the extended Cohn-Kanade dataset to compare three object recognition techniques: linear support vector classification, linear discriminant analysis and softmax. More than 90% performance rates, with low standard deviations, were achieved by Boonsuk et al. [14]. The use of manpower is difficult in case of natural disaster occurrence in hilly areas, and continuous electric power supply is highly affected in these areas due to maintenance issues of transmission lines. Therefore, in this case autopilot aerial equipment is used to gather images, and hidden content from aerial images needs to be identified in case of natural disasters such as landslides and heavy snowfall. Zhou et al. [15] removed the noise from raw aerial images and extracted disaster characteristics using the interframe difference technique; they implemented a convolutional neural network to analyze the type of disaster. In some regions, disasters such as earthquakes are inclined to occur due to geographical factors. To locate the victim in a short time is crucial; Sulistijono et al. [16] acquired aerial images, and locating the victims was made possible by using a dedicated ground station server and proposed victim detection framework based on convolution neural networks. A simulation of real calamities was developed to test the framework.

Floods are a calamitous and remarkable disaster. Floods impact greatly on human lives, economically and financially affecting nations. With the help of a neural network, it is possible to predict floods and save the masses from the disaster. By implementing a convolutional neural network and Modified Particle Swarm Optimization (MPSO), Padmawar et al. [17] developed a deep learning approach to foresee the flood circumstances and identify the individuals beforehand. 

Chen et al. [18] proposed unmanned aerial vehicle image-based forest fire detection images of forest fires, stabilized the histogram and applied filters to smoothen the images before testing via convolutional neural network. Smoke detection was carried out using the local binary pattern (LBP) and support vector machine (SVM). Comparison of processed and raw images was made to test the effectiveness of the proposed strategy. 

Forest fires drastically affect human lives and economic situations, and locating the victims in a short time is complex task. Convolutional neural networks make it possible to help firefighters to locate the location of victims by detecting density of smoke from images acquired from the unmanned aerial vehicle. CNN-based simple feature extraction with a AlexNet single deconvolution (SFEwAN-SD)-based proposed approach helps develop a real time fire monitoring system (Gonzalez et al. [19]). Samudre et al. [20] successfully improved response time, reduced power consumption, and optimized performance by using pipelining among network layers of a CNN, executed on a field-programmable gate array. As the spatial resolution of satellite images was too low, these images could not be used for wildfire detection; Lee et al. [21] modified deep convolutional networks for high spatial resolution images, VGG-13 and Google Net, utilizing UAVs, a disaster forecasting system, web-based visualization system, alert system, and disaster response scenario database and achieved highly accurate results for early wildfire detection. It is a hectic job for a disaster management organization to assess the damage caused by natural disasters. Using images obtained from social media during and after the occurrence of four major natural disasters, Nguyen et al. [22] proposed a method by adapting CNN features based on event-specific and cross-events. Direkoglu et al. [23] proposed a method to produce motion information images computing optical flow vectors and employed a CNN; the proposed method efficiently differentiated normal and abnormal behaviors of people during a natural disaster. UMN and PETS2009 datasets were used to performed experiments. Yuan et al. [24] proposed a wave-shaped neural network (W-Net) to label the density of smoke in images, which is difficult task, so virtual dataset was created. Convolutional encoder decoder architectures were assembled to maximize the input for information extraction from smoke density images and W-Net was proposed. The accuracy of the proposed system is improved by feeding previous encoding outputs to the decoding layers and combining them. Several data mining application were implemented using contents of social media; user generated content helps in disastrous events to gain vast amount of information. The CNN model is used to extract flood images from raw images and color filters are used to refine the desired detection. In the work of Layek et al. [25], the proposed system’s efficiency and accuracy were tested on several datasets and it outperformed other methods to give the highest results. The proposed multilayered convolutional neural network in this research is used to detect and classify the natural disasters, as explained in the methodology section. Moreover, a comparison of the some of the state-of-the-art methods is shown in Table 1.

## 3. Methodology

**Figure 1:**
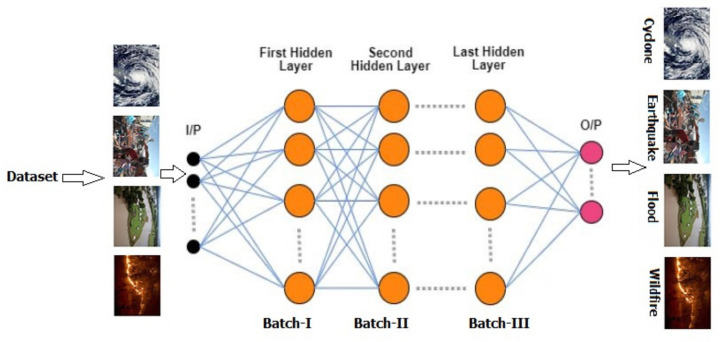
Proposed architecture of multilayered deep convolutional neural network.

This section defines the overall method for natural disaster intensity analysis and classification based on multispectral images using a multilayered deep convolutional neural network. Moreover, this method consists of two blocks of a convolutional neural network. The first block detects a natural disaster occurring and the second one defines the intensity type of the natural disaster. Additionally, the first block consists of three miniconvolutional blocks with four layers each, including an image input and fully connected layers. On the other hand, the second block also consists of three miniconvolutional blocks with two layers each and includes an image input layer and fully connected layer. The overall flow of methodology is shown in Figure 1 and explained below.

### 3.1. Block-I Convolutional Neural Network (B-I CNN)

**Figure 2:**
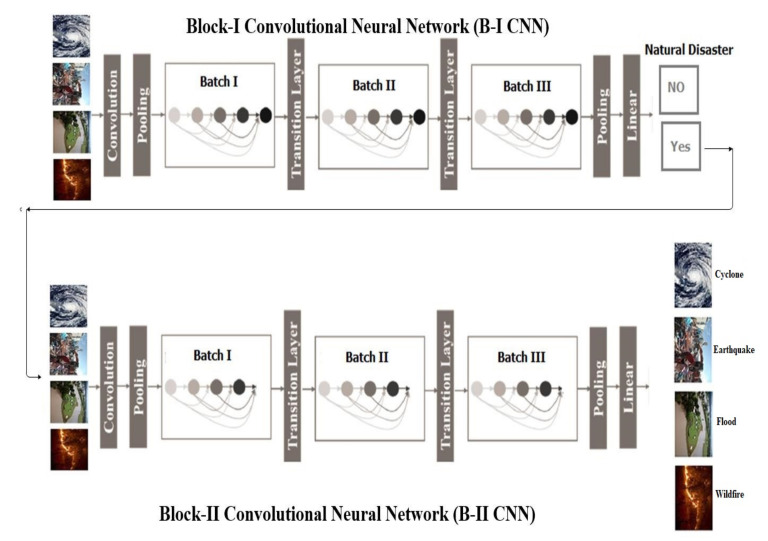
Architecture of proposed multilayered deep convolutional neural network.

According to block-I of the convolutional neural network, only a detection process occurred in this phase. However, this block also consists of three small batches having four layers each. Moreover, an image input layer and fully connected layers are present. Additionally, some parameters are also defined with learning rate 0.001 and epoch size 40. On the other hand, the convolutional layers use a filter size of 3 × 3, stride 1 and eight filters that increase in number from 16 to 32 for the second and third minibatches of convolutional neural networks, as shown in Table 2 and Figure 2.

### 3.2. Block-II Convolutional Neural Network (B-II CNN)

The block-II convolutional neural network takes the output from the first block and finds the types of natural disaster with intensity. Moreover, this block also consists of three minibatches having three layers each with two extra layers such as image input and fully connected layers. Additionally, the same parameters as block-I have been defined for this block also. The description of parameters is given in Table 3 and Figure 2.

## 4. Results and Discussion

The proposed multilayered deep convolutional neural network was simulated on the computer system with Core i7, Central Processing Unit (CPU) 2.8 Ghz with 16 GB RAM in MATLAB 2018a and different types of results were calculated. 

### 4.1. Dataset and Preprocessing

**Figure 3:**
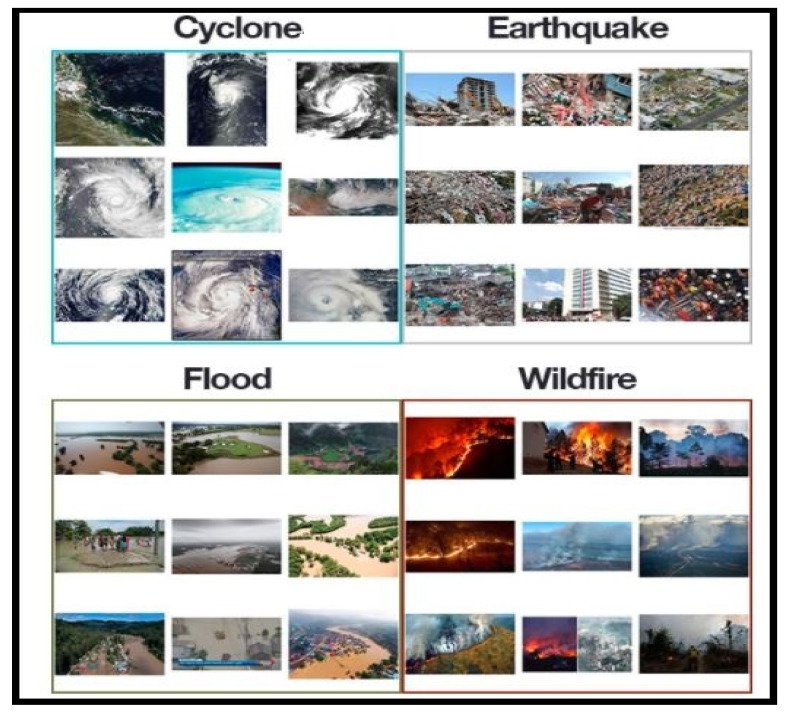
Different classes of natural disasters from dataset.

In our research, the dataset used was collected from PyImage Search readers, who used Google Images to collect the total number (4428) of images in different classes. The dataset was separated into four classes: cyclone, earthquake, flood and wildfire, with 928, 1350, 1073 and 1077 images, respectively, as shown in Figure 3. The dataset was preprocessed to remove the noise by using an adaptive histogram equalizer. The whole dataset was divided into three groups: training, testing and validation. In total, 60% of the dataset was used for training, 23% for testing and 17% for validation. These percentages of the dataset were used to inform the machine on the percentage values of the dataset to be used for testing, training and validation purposes. The validation set was used to count the number of epochs for the whole training process. The details of the dataset are shown in Table 4.

### 4.2. Evaluation Criterion

**Figure 4:**
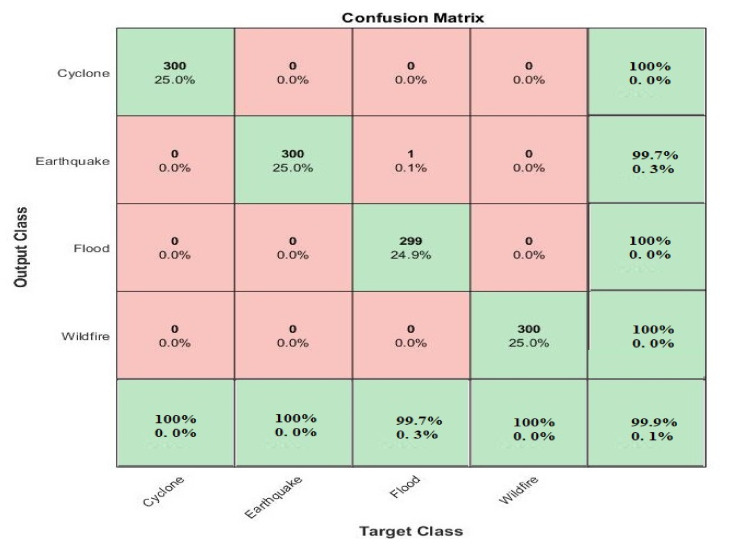
4-Class matrix of natural disasters classification by using the proposed method on the testing dataset.

**Figure 5:**
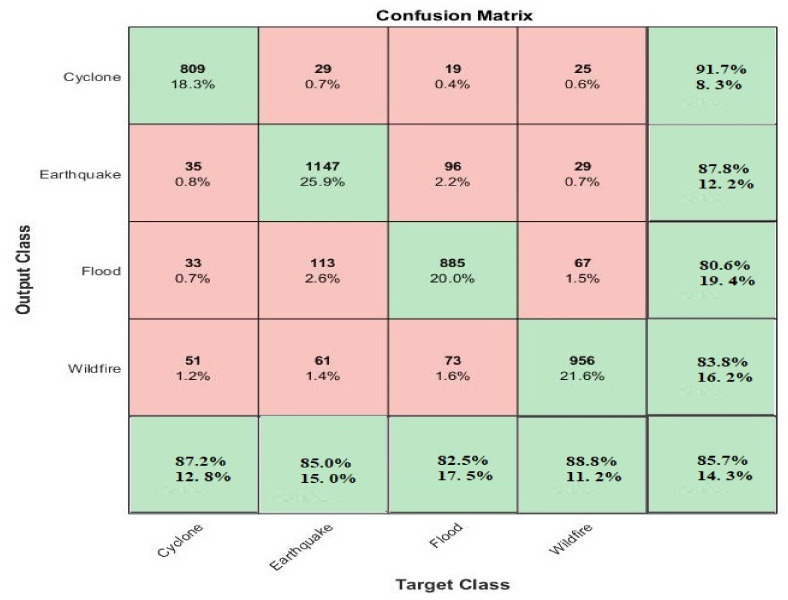
Confusion matrix of 4-class of natural disaster classification by using the proposed method on the training dataset.

To evaluate the performance of the proposed multilayered deep convolutional neural network, uses a train–test validation schema. To train the whole model, the training dataset was used, while for the fine-tuning of model the validation set was used. The performance of the whole framework was calculated on the basis of the test dataset. For the evaluation of the proposed model on the given dataset of classification for positive and negative values, four types of data were accrued: true positive (TP), the number of correctly positive classified images; true negative (TN) the number of correctly negative classified images; false positive (FP), the number of incorrectly positive classified images; and false negative (FN), the number of images that are incorrectly classified as negative images. The confusion matrices for these values are shown in Figure 4 and Figure 5. To calculate the performance of the model, the specificity (SP), sensitivity (SE), accuracy rate (RR), precision (PRE) statistical values were adopted as a criteria. The F1 score was used when a conflict occurred between accuracy and sensitivity to evaluate the performance. The equations are given below.

(1) $$Sensitivity\;\left(SE\right)\;=\frac{TP}{TP+FN}$$

The sensitivity (*SE*) in Equation (1) is the true positive measurement, the ratio of correctly identified values.

(2) $$Specificity\;\left(SP\right)\;=\frac{TN}{TP+FP}$$

Equation (2) shows the value of specificity (*SP*), the ratio of negatives which are correctly classified.

(3) $$Accuracy\;Rate\;\left(AR\right)\;=\frac{TP+TN}{TP+TN+FP+FN}$$

Equation (3) gives the value of accuracy rate (*AR*), which is equal to the actual measurement of specified values.

(4) $$Precision\;\left(PRE\right)\;=\;\frac{TP}{TP+FP}$$

The precision (*PRE*) in Equation (4) explains the proportion of closeness in measurement values.

(5) $$F1-Score\;\left(F1\right)\;=\;\frac{2\left(SE\;\times \;PRE\right)}{SE+\;PRE}$$

The *F*1–Score (*F*1) in Equation (5) is the proportion of recall and precision which actually measure the model accuracy for the dataset.

**Figure 6:**
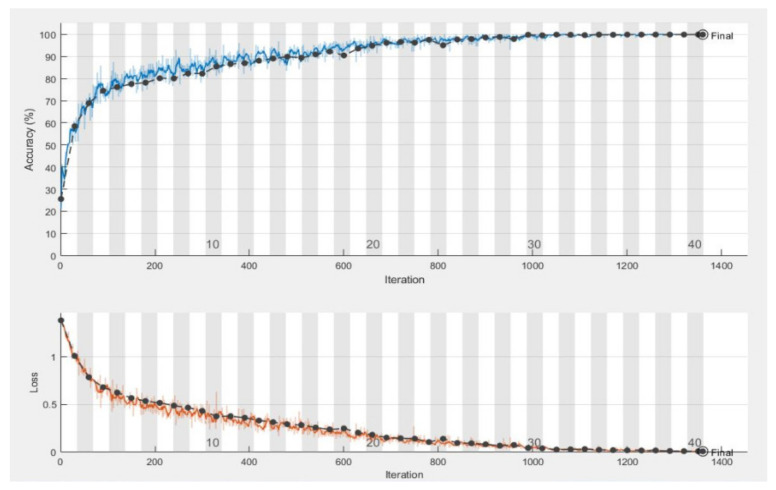
Graphical representation of training and validation accuracy and loss on various iterations.

The graph in Figure 6 shows the training and validation accuracy rate, which is 99.92%, and also shows the validation and training loss. Moreover, a complete training process is represented in Figure 6. The smooth line shows the training process and the dotted line shows the validation process for natural disasters dataset. Table 5 shows the calculated results in the shape of average statistical values: SE, 97.54%; SP, 98.22%; AR, 99.92%; PRE, 97.79%; and F1, 97.97% for the proposed model. The obtained results are comparable with the state-of-the-art techniques and solved the complex queries related to analysis of the natural disasters.

The overall comparison of results with the state-of-the-art methods is shown in Table 6. The proposed model shows better accuracy as compared to the recently developed techniques. The reason for this is that the proposed technique works in two parts: one for natural disaster occurrence detection and the second one for natural disaster classifications. The overall proposed model works on an image dataset to detect and classify the natural disasters. As the model is evaluated on a simple central processing unit (CPU)-based system, it only detects disaster types and then classifies them into cyclone, earthquake, flood and wildfire classes. However, if this model is run on a graphic processing unit (GPU)-based system in the future with real time sensors and monitoring power, then the proposed model will be used as a real time natural disaster detection model and provide some upcoming predictions for future disasters. The main purpose of this model is to detect and classify the type of disaster with a high accuracy rate. To prevent natural disasters in the future, said model can be used to predict future disasters and take some action against heavy loss of human ecological systems and property. 

## 5. Conclusions

Many researchers have attempted to use different deep learning methods for detection of natural disasters. However, the detection of natural disasters by using deep learning techniques still faces various issues due to noise and serious class imbalance problems. To address these problems, we proposed a multilayered deep convolutional neural network for detection and intensity classification of natural disasters. The proposed method works in two blocks—one for detection of natural disaster occurrence and the second block is used to remove imbalanced class issues. The results were calculated as average statistical values: sensitivity, 97.54%; specificity, 98.22%; accuracy rate, 99.92%; precision, 97.79%; and F1-score, 97.97% for the proposed model. The proposed model achieved the highest accuracy as compared to other state-of-the-art methods due to its multilayered structure. The proposed model performs significantly better for natural disaster detection and classification, but in the future the model can be used for various natural disaster detection processes.

## Figures and Tables

**Table 1:** Comparison of state-of-the-art techniques.

Reference	Methodology Name	Outcomes	Weakness
[26]	Signal processing, image processing and statistical technique	More accurate prediction of natural disasters	Limited statistical parameters for prediction
[27]	Particle swarm optimization	Predict magnitude of earthquake	Work only for prediction on seismic dataset
[28]	Neural network	Predict magnitude of earthquake	Limited parameters used for prediction
[29]	Text mining, regular log mining technique	Detect earthquake with speed and accuracy on seismological data	Depends on public feedback to detect earthquake
[30]	Decision tree	Utilize some parameters to access the model for flood damage area detection	Parametric limitation for the detection of flood damaging regions
[31]	Artificial neural network, genetic algorithm and wavelet transfer technique	Sum-up good results as compared to the already existing techniques in the southeast Asia	Work for monsoon floods in June and September for specific regions in India for time series data
[32]	Support vector machine, naïve Bayes	Classify the natural disasters on various parameters	Limited for only early stages of natural disasters
[33]	Machine learning technique	Predict the land slidding with the accuracy rate of 75 to 95	More guidlines for model selection for predition large scale landslide
[34]	Neural network and back propagation	Prediction occur on past dataset	Dyanamic prediction is very much crucial for this system
[35]	Clustering for multivariable time series	Proposed a dynamic clustering approch for time series analysis and self-optimize organizing mapping technique	Dynamic time series data required for clustering process
[36]	Data mining technique	A real time desktop-based GUI system is designed to predict local storm	Use parallel computing process that takes various amounts of time to predict storm
[37]	Text mining technique	Develop a public platform to inform early tsunami prediction and information	Public feedback is compulsory for prediction process
[38]	Random forest, long short-term model	Evaluate the flood severity in terms of sensitivity, specificity and accuracy as 71.4%, 85.9%, 81.13%, respectively	Particle swarm optimization and other deep learning techniques can be used as a future work
[39]	A learning-based wildfire model	Proposed method can predict the short term spread of wildfire	Real time rate of wildfire spread is required for initial stage
[40]	Machine learning technique	The gradient boosting tree and CLIPER model used for cyclone prediction	Model is still weak to produce velocity sensitivities
[41]	Machine learning technique with numerical weather prediction	The prediction method is used for China that shows significant improvement as compared to the traditional methods	Still lack symmetric parameters for numerical computations
[42]	Artificial neural network	A fully connected neural network for segmentation which is used for multivariable pattern recognition at different levels	It works on multivariable parameters rather than the pixel by pixel parameters

**Table 2:** Block-I Convolutional Neural Network (B-I CNN).

Block-I Convolutional Neural Network (B-I CNN) with Learning Rate = 0.001 and Epochs = 40		
Layer Name and Batches		Parameters
	Image Input Layer	Height: 100, Width: 120, Channel: 3
**Batch I:**	Convolution Layer Batch Normalization Layer Relu Layer Max Pooling Layer	Filter size: 3 × 3, No. of filters = 8, stride = 1
**Batch II:**	Convolution Layer Batch Normalization Layer Relu Layer Max Pooling Layer	Filter size: 3 × 3, No. of filters = 16, stride = 1
**Batch III:**	Convolution Layer Batch Normalization Layer Relu Layer Max Pooling Layer	Filter size: 3 × 3, No. of filters = 32, stride = 1
	Fully Connected Layer	4 Classes

**Table 3:** Block-II convolutional neural network (B-II CNN).

Block-II Convolutional Neural Network (B-II CNN) with Learning Rate = 0.001 and Epochs = 30		
Layer Name and Batches		Parameters
	Image Input Layer	Height: 100, Width: 120, Channel: 3
**Batch I:**	Convolution Layer Batch Normalization Layer Max Pooling Layer	Filter size: 3 × 3, No. of filters = 8, stride = 1
**Batch II:**	Convolution Layer Batch Normalization Layer Max Pooling Layer	Filter size: 3 × 3, No. of filters = 16, stride = 1
**Batch III:**	Convolution Layer Batch Normalization Layer Max Pooling Layer	Filter size: 3 × 3, No. of filters = 32, stride = 1
	Fully Connected Layer	4 Classes

**Table 4:** Grouping of natural disasters dataset.

Disaster Type	Total	Training	Test	Validation
Cyclone	928	500	300	128
Earthquake	1350	600	300	450
Flood	1073	600	300	173
Wildfire	1077	600	300	177
Total	4428	2300	1200	928

**Table 5:** Statistical value calculations of proposed model for the whole dataset.

Sr.	Disaster Type	SE (%)	SP (%)	AR (%)	PRE (%)	F1 (%)
1	Cyclone	97.15	98.08	100.00	97.32	97.36
2	Earthquake	95.18	97.11	99.70	96.34	98.88
3	Flood	99.17	99.13	100.00	99.05	99.23
4	Wildfire	98.67	98.56	100.00	98.45	96.44
Average		97.54	98.22	99.92	97.79	97.97

**Table 6:** State-of-the-art comparison of the proposed multilayered deep convolutional neural network.

Cited	Technique Used	Accuracy-Rate (%)	Year
[43]	CNN	84.00	2015
[44]	Feed-Forward neural network	92.00	2016
[45]	Support Vector Machine	87.00	2016
[46]	CNN	90.00	2016
[47]	Glaucoma-Deep (CNN, DBN d, Softmax)	99.0	2017
[48]	RestNet-50	96.02	2018
[7]	WSDD-Net	99.20	2019
[49]	OCT Probability map using CNN	95.7	2019
[50]	Attention Guided Convolutional Neural Network	95.3	2019
[51]	ML-DCNN	99.39	2020
[52]	ML-DCNNet	99.14	2020
Proposed Multilayered Deep Convolutional Neural Network		99.92	2021

## Data Availability

The results were obtained by using the following publicly available dataset: https://drive.google.com/file/d/1NvTyhUsrFbL91E10EPm38IjoCg6E2c6q/view (accessed on 25 March 2021).

## References

[1] Mignan A., Broccardo M. (2020). Neural network applications in earthquake prediction (1994–2019): Meta-analytic and statistical insights on their limitations. Seism. Res. Lett..

[2] Tonini M., D’Andrea M., Biondi G., Degli Esposti S., Trucchia A., Fiorucci P. (2020). A Machine Learning-Based Approach for Wildfire Susceptibility Mapping. The Case Study of the Liguria Region in Italy. Geosciences.

[3] Islam A.R.M.T., Talukdar S., Mahato S., Kundu S., Eibek K.U., Pham Q.B., Kuriqi A., Linh N.T.T. (2021). Flood susceptibility modelling using advanced ensemble machine learning models. Geosci. Front..

[4] Schlemper J., Caballero J., Hajnal V., Price A.N., Rueckert D. (2017). A deep cascade of convolutional neural networks for dynamic MR image reconstruction. IEEE Trans. Med. Imaging.

[5] Tang C., Zhu Q., Wu W., Huang W., Hong C., Niu X. (2020). PLANET: Improved convolutional neural networks with image enhancement for image classification. Math. Probl. Eng..

[6] Ashiquzzaman A., Oh S.M., Lee D., Lee J., Kim J. (2021). Context-aware deep convolutional neural network application for fire and smoke detection in virtual environment for surveillance video analysis. Smart Trends in Computing and Communications, Proceedings of the SmartCom 2020, Paris, France, 29–31 December 2020.

[7] Li T., Zhao E., Zhang J., Hu C. (2019). Detection of Wildfire Smoke Images Based on a Densely Dilated Convolutional Network. Electronics.

[8] Mangalathu S., Burton H.V. (2019). Deep learning-based classification of earthquake-impacted buildings using textual damage descriptions. Int. J. Disaster Risk Reduct..

[9] Hartawan D.R., Purboyo T.W., Setianingsih C. Disaster Victims Detection System Using Convolutional Neural Network (CNN) Method. Proceedings of the 2019 IEEE International Conference on Industry 4.0, Artificial Intelligence, and Communications Technology (IAICT).

[10] Amit S.N.K.B., Aoki Y. Disaster detection from aerial imagery with convolutional neural network. Proceedings of the 2017 International Electronics Symposium on Knowledge Creation and Intelligent Computing (IES-KCIC).

[11] Yang S., Hu J., Zhang H., Liu G. (2021). Simultaneous Earthquake Detection on Multiple Stations via a Convolutional Neural Network. Seism. Res. Lett..

[12] Madichetty S., Sridevi M. Detecting informative tweets during disaster using deep neural networks. Proceedings of the 2019 11th International Conference on Communication Systems & Networks (COMSNETS).

[13] Nunavath V., Goodwin M. The role of artificial intelligence in social media big data analytics for disaster management-initial results of a systematic literature review. Proceedings of the 2018 5th International Conference on Information and Communication Technologies for Disaster Management (ICT-DM).

[14] Boonsuk R., Sudprasert C., Supratid S. An Investigation on Facial Emotional Expression Recognition Based on Linear-Decision-Boundaries Classifiers Using Convolutional Neural Network for Feature Extraction. Proceedings of the 2019 11th International Conference on Information Technology and Electrical Engineering (ICITEE).

[15] Zhou F., Huang J., Sun B., Wen G., Tian Y. Intelligent Identification Method for Natural Disasters along Transmission Lines Based on Inter-Frame Difference and Regional Convolution Neural Network. Proceedings of the 2019 IEEE International Conference on Parallel & Distributed Processing with Applications, Big Data & Cloud Computing, Sustainable Computing & Communications, Social Computing & Networking (ISPA/BDCloud/SocialCom/SustainCom).

[16] Sulistijono I.A., Imansyah T., Muhajir M., Sutoyo E., Anwar M.K., Satriyanto E., Basuki A., Risnumawan A. Implementation of Victims Detection Framework on Post Disaster Scenario. Proceedings of the 2018 International Electronics Symposium on Engineering Technology and Applications (IES-ETA).

[17] Padmawar P.M., Shinde A.S., Sayyed T.Z., Shinde S.K., Moholkar K. Disaster Prediction System using Convolution Neural Network. Proceedings of the 2019 International Conference on Communication and Electronics Systems (ICCES).

[18] Chen Y., Zhang Y., Xin J., Wang G., Mu L., Yi Y., Liu H., Liu D. UAV Image-based Forest Fire Detection Approach Using Convolutional Neural Network. Proceedings of the 2019 14th IEEE Conference on Industrial Electronics and Applications (ICIEA).

[19] Gonzalez A., Zuniga M.D., Nikulin C., Carvajal G., Cardenas D.G., Pedraza M.A., Fernández C., Munoz R., Castro N., Rosales B. Accurate fire detection through fully convolutional network. Proceedings of the 7th Latin American Conference on Networked and Electronic Media (LACNEM 2017).

[20] Samudre P., Shende P., Jaiswal V. Optimizing Performance of Convolutional Neural Network Using Computing Technique. Proceedings of the 2019 IEEE 5th International Conference for Convergence in Technology (I2CT).

[21] Lee W., Kim S., Lee Y.-T., Lee H.-W., Choi M. Deep neural networks for wild fire detection with unmanned aerial vehicle. Proceedings of the 2017 IEEE International Conference on Consumer Electronics (ICCE).

[22] Nguyen D.T., Ofli F., Imran M., Mitra P. Damage assessment from social media imagery data during disasters. Proceedings of the 2017 IEEE/ACM International Conference on Advances in Social Networks Analysis and Mining.

[23] Direkoglu C. (2020). Abnormal Crowd Behavior Detection Using Motion Information Images and Convolutional Neural Networks. IEEE Access.

[24] Yuan F., Zhang L., Xia X., Huang Q., Li X. (2019). A wave-shaped deep neural network for smoke density estimation. IEEE Trans. Image Process..

[25] Layek A.K., Poddar S., Mandal S. Detection of Flood Images Posted on Online Social Media for Disaster Response. Proceedings of the 2019 Second International Conference on Advanced Computational and Communication Paradigms (ICACCP).

[26] Amezquita-Sanchez J., Valtierra-Rodriguez M., Adeli H. (2017). Current efforts for prediction and assessment of natural disasters: Earthquakes, tsunamis, volcanic eruptions, hurricanes, tornados, and floods. Sci. Iran..

[27] Zhang X.Y., Li X., Lin X. (2014). The data mining technology of particle swarm optimization algorithm in earthquake prediction. Adv. Mater. Res..

[28] Adeli H., Panakkat A. (2009). A probabilistic neural network for earthquake magnitude prediction. Neural Netw..

[29] Kradolfer U. SalanderMaps: A rapid overview about felt earthquakes through data mining of web-accesses. Proceedings of the EGU General Assembly Conference.

[30] Merz B., Kreibich H., Lall U. (2013). Multi-variate flood damage assessment: A tree-based data-mining approach. Nat. Hazards Earth Syst. Sci..

[31] Sahay R.R., Srivastava A. (2014). Predicting monsoon floods in rivers embedding wavelet transform, genetic algorithm and neural network. Water Resour. Manag..

[32] Venkatesan M., Thangavelu A., Prabhavathy P. An improved Bayesian classification data mining method for early warning landslide susceptibility model using GIS. Proceedings of the Seventh International Conference on Bio-Inspired Computing: Theories and Applications (BIC-TA 2012).

[33] Korup O., Stolle A. (2014). Landslide prediction from machine learning. Geol. Today.

[34] Di Salvo R., Montalto P., Nunnari G., Neri M., Puglisi G. (2013). Multivariate time series clustering on geophysical data recorded at Mt. Etna from 1996 to 2003. J. Volcanol. Geotherm. Res..

[35] Das H.S., Jung H. (2013). An efficient tool to assess risk of storm surges using data mining. Coast. Hazards.

[36] Chatfield A.T., Brajawidagda U. Twitter early tsunami warning system: A case study in Indonesia’s natural disaster management. Proceedings of the 2013 46th Hawaii International Conference on System Sciences.

[37] Khalaf M., Alaskar H., Hussain A.J., Baker T., Maamar Z., Buyya R., Liatsis P., Khan W., Tawfik H., Al-Jumeily D. (2020). IoT-enabled flood severity prediction via ensemble machine learning models. IEEE Access.

[38] Zhai C., Zhang S., Cao Z., Wang X. (2020). Learning-based prediction of wildfire spread with real-time rate of spread measurement. Combust. Flame.

[39] Tan J., Chen S., Wang J. (2020). Western North Pacific tropical cyclone track forecasts by a machine learning model. Stoch. Environ. Res. Risk Assess..

[40] Liu Y.Y., Li L., Liu Y.S., Chan P.W., Zhang W.H., Zhang L. (2021). Estimation of precipitation induced by tropical cyclones based on machine-learning-enhanced analogue identification of numerical prediction. Meteorol. Appl..

[41] Meadows M., Wilson M. (2021). A Comparison of Machine Learning Approaches to Improve Free Topography Data for Flood Modelling. Remote Sens..

[42] Nisa A.K., Irawan M.I., Pratomo D.G. (2019). Identification of Potential Landslide Disaster in East Java Using Neural Network Model (Case Study: District of Ponogoro). J. Phys. Conf. Ser..

[43] Chen X., Xu Y., Wong D.W.K., Wong T.Y., Liu J. Glaucoma detection based on deep convolutional neural network. Proceedings of the 2015 37th Annual International Conference of the IEEE Engineering in Medicine and Biology Society (EMBC).

[44] Asaoka R., Murata H., Iwase A., Araie M. (2016). Detecting preperimetric glaucoma with standard automated perimetry using a deep learning classifier. Ophthalmology.

[45] Salam A.A., Khalil T., Akram M.U., Jameel A., Basit I. (2016). Automated detection of glaucoma using structural and non structural features. Springerplus.

[46] Claro M., Santos L., Silva W., Araújo F., Moura N., Macedo A. (2016). Automatic glaucoma detection based on optic disc segmentation and texture feature extraction. CLEI Electron. J..

[47] Abbas Q. (2017). Glaucoma-deep: Detection of glaucoma eye disease on retinal fundus images using deep learning. Int. J. Adv. Comput. Sci. Appl..

[48] Li Y., Xie X., Shen L., Liu S. (2019). Reverse active learning based atrous DenseNet for pathological image classification. BMC Bioinform..

[49] Thakoor K.A., Li X., Tsamis E., Sajda P., Hood D.C. Enhancing the Accuracy of Glaucoma Detection from OCT Probability Maps using Convolutional Neural Networks. Proceedings of the 2019 41st Annual International Conference of the IEEE Engineering in Medicine and Biology Society (EMBC).

[50] Li L., Xu M., Wang X., Jiang L., Liu H. Attention Based Glaucoma Detection: A Large-scale Database and CNN Model. Proceedings of the Proceedings of the IEEE Conference on Computer Vision and Pattern Recognition.

[51] Aamir M., Irfan M., Ali T., Ali G., Shaf A., Al-Beshri A., Alasbali T., Mahnashi M.H. (2020). An Adoptive Threshold-Based Multi-Level Deep Convolutional Neural Network for Glaucoma Eye Disease Detection and Classification. Diagnostics.

[52] Aamir M., Ali T., Shaf A., Irfan M., Saleem M.Q. (2020). ML-DCNNet: Multi-level Deep Convolutional Neural Network for Facial Expression Recognition and Intensity Estimation. Arab. J. Sci. Eng..

